# Plasma metformin concentration as a determinant of metabolic response in adults with type 1 diabetes: a 1-year prospective observational study

**DOI:** 10.3389/fphar.2026.1854936

**Published:** 2026-06-30

**Authors:** Małgorzata Bekier, Aleksandra Uruska, Michał Michalak, Andrzej Czyrski, Maja Miętkiewska-Dolecka, Danuta Szkutnik-Fiedler

**Affiliations:** 1 Department of Clinical Pharmacy and Biopharmacy, Poznań University of Medical Sciences, Poznan, Poland; 2 Department of Internal Medicine and Diabetology, Poznan University of Medical Sciences, Poznan, Poland; 3 Department of Computer Science and Statistics, Poznan University of Medical Sciences, Poznan, Poland; 4 Department of Physical Pharmacy and Pharmacokinetics, Poznan University of Medical Sciences, Poznan, Poland; 5 Department of Internal Medicine and Diabetology, Doctoral School, Poznan University of Medical Sciences, Poznan, Poland

**Keywords:** cardiometabolic risk, eGDR, insulin resistance, metformin, pharmacokinetics, time in range, type 1 diabetes

## Abstract

**Background:**

Metformin is commonly used as adjunctive therapy in adults with type 1 diabetes (T1DM), although evidence regarding its long-term metabolic effects remains inconsistent. To assess the metabolic effects of adjunctive metformin therapy in adults with type 1 diabetes (T1DM) over a 12-month period, using advanced markers of metabolic control, including time in range (TIR), estimated glucose disposal rate (eGDR), and monitoring of metformin plasma concentrations.

**Methods:**

This single-center, observational study included 26 adults with T1DM treated with extended-release metformin (500–2000 mg/day). Metformin plasma concentrations were measured at baseline and at 5 time points up to 12 months using a validated UPLC-MS/MS method. Glycemic control (TIR), insulin dose, anthropometric parameters, lipid profile, visceral adiposity index (VAI), and eGDR were assessed. Participants were grouped by median metformin concentration.

**Results:**

Interindividual variability in metformin plasma concentrations was significant. Adjunctive metformin therapy was associated with significant reductions in LDL cholesterol, non-HDL cholesterol, and VAI. A significant transient improvement in eGDR was observed after 6 months of therapy, followed by partial attenuation at 12 months. A significant interaction between metformin concentration subgroup and time was observed for TIR (p = 0.0151), with descriptively greater improvement in participants with higher metformin concentrations. No statistically significant differences in metabolic response were identified between subgroups with lower and higher mean metformin concentrations.

**Conclusion:**

Adjunctive metformin therapy in adults with T1DM was associated with favorable changes in insulin sensitivity markers and cardiometabolic risk parameters during follow-up. Monitoring metformin plasma concentrations and eGDR may provide complementary information for metabolic assessment during adjunctive metformin therapy in adults with T1DM.

## 1 Introduction

Type 1 diabetes mellitus (T1DM) is a chronic autoimmune disease resulting from the destruction of pancreatic β cells. This results in complete insulin deficiency and the need for lifelong exogenous insulin therapy. Intensive insulin therapy can be administered through multiple daily injections (MDI) or continuous subcutaneous insulin infusion (CSII). The main goal of treating T1DM is to achieve a state close to normoglycemia while minimizing the risk of hypoglycemia and long-term cardiovascular complications (American Diabetes Association, 2024). Despite advances in insulin formulations, delivery systems, and continuous glucose monitoring technology, achieving optimal metabolic control remains a challenge in the management of T1DM (Bielka et al., 2024).

In some adults with T1DM, we also observe insulin resistance characteristics, typical of type 2 diabetes, such as visceral obesity, dyslipidemia, and hypertension. As described by Cleland et al. (2013), insulin resistance in T1DM may occur independently of autoimmune mechanisms and is strongly dependent on environmental and metabolic factors, including weight gain and lifestyle. High doses of exogenous insulin used in intensive insulin therapy may lead to peripheral hyperinsulinemia, thereby increasing insulin resistance and insulin demand. As a result, double diabetes (DD) significantly modifies the clinical course of T1DM and is associated with an increased risk of cardiovascular complications, despite the preserved autoimmune etiology (Cleland et al., 2013; Petrie et al., 2017). The common practice of pharmacological treatment for DD is to combine intensive insulin therapy with drugs typically used for type 2 diabetes. The most common is metformin (MET). This drug is not officially indicated for use in adults with type 1 diabetes, but it may improve insulin sensitivity in this population. In addition, they may affect components of the metabolic syndrome, such as blood pressure or lipid profile (Bielka et al., 2024; Petrie et al., 2017; Timmons et al., 2021; Meng et al., 2018; Liu et al., 2020; Langford et al., 2020; Xu et al., 2022; Vella et al., 2010).

Metformin is a member of the biguanide class of drugs, which has a pleiotropic mechanism of action. Its primary clinical effect is to decrease blood glucose levels by reducing glucose production in the liver and improving peripheral tissue sensitivity to insulin. At the molecular level, metformin inhibits the mitochondrial respiratory chain in the liver, leading to the activation of AMPK, which enhances insulin sensitivity (*via* effects on fat metabolism) and lowers cAMP, thereby reducing the expression of gluconeogenic enzymes. Metformin also has AMPK-independent effects on the liver which may include the inhibition of fructose-1,6-bisphosphatase by AMP (Rena et al., 2017). Highlighted that despite wide knowledge about the mechanism of action of metformin, we still need research results based on long-term oral use of this drug in specific populations. A reliable assessment of the clinical effect may depend not only on the dose of metformin, but also on the duration of treatment (Petrie et al., 2017; Timmons et al., 2021; Rena et al., 2017).

Insulin resistance has emerged as a significant determinant of cardiovascular and microvascular risk in T1DM (Orchard et al., 2003; Chen et al., 2023; Bjornstad et al., 2018). The hyperinsulinemic-euglycemic clamp remains the gold standard for assessing insulin sensitivity. However, its complexity and limited feasibility in routine clinical practice have prompted the development of surrogate indices (Williams et al., 2000). The estimated glucose disposal rate (eGDR) is a validated, non-invasive measure of insulin sensitivity derived from waist circumference, hypertension status, and HbA1c (Chen et al., 2023; Williams et al., 2000; Cree-Green et al., 2019).

The eGDR is a validated clinical tool for estimating insulin sensitivity in people with T1DM, providing useful information beyond traditional measures. Lower eGDR levels, indicating greater insulin resistance, are associated with a higher prevalence of vascular complications in people with T1DM (Epstein et al., 2013; Urus et al., 2010). Therefore, a combined assessment of TIR and eGDR may provide a more comprehensive evaluation of the metabolic effects of metformin therapy than conventional glycemic indicators alone. The use of eGDR may be particularly relevant in the context of adjunctive metformin therapy, where metabolic benefits may precede changes in mean glycemia or occur independently of them (Petrie et al., 2017; Poh et al., 2023).

Therefore, the objective of this study was to evaluate the metabolic effects of adjunctive metformin therapy in adults with T1DM over a 1-year observation period using advanced markers of metabolic control. Treatment efficacy was assessed with particular emphasis on time in range (TIR) and estimated glucose disposal rate (eGDR), alongside conventional anthropometric and lipid parameters. Longitudinal monitoring of plasma metformin concentrations was performed to accurately characterize treatment exposure and adherence, addressing a key limitation of previous long-term studies.

The primary exploratory outcomes of the study were longitudinal changes in insulin sensitivity assessed by eGDR and glycemic control assessed by TIR during adjunctive metformin therapy. Secondary outcomes included changes in insulin dose, lipid profile, anthropometric parameters, VAI, and plasma metformin concentrations. ANN-derived analyses were predefined as exploratory and hypothesis-generating.

## 2 Materials and methods

### 2.1 Study design

The study comprised 26 adults with T1DM who received metformin as an adjunct therapy. Inclusion criteria were: type 1 diabetes confirmed with autoantibodies, ≥18 years old, written consent to participate in the study. Exclusion criteria included renal (eGFR <30 mL/min/1.73 m^2^) and liver failure, as well as individuals whose condition, in the opinion of a medical professional, disqualified them from participating in the study.

This prospective real-world observational study was designed to reflect routine clinical management of adults with T1DM receiving adjunctive metformin therapy. Treatment decisions, including metformin dose adjustments, insulin titration, glucose monitoring modalities, and concomitant pharmacotherapy, were individualized according to routine clinical practice rather than predefined interventional protocols. Participants continued their standard diabetes care throughout the study period under non-randomized treatment conditions.

Participants received a prescription and self-administered the medication at home. Participants were instructed by a pharmacist on how to take the medication correctly. All experimental protocols were approved by the Bioethics Committee (No 825/21). All research was performed in accordance with relevant regulations. The material for the study involved blood samples collected at the following time points: 1 day (visit 1), 14 days (visit 2), 1 month (visit 3), 3 months (visit 4), 6 months (visit 5), and 1 year (visit 6) of the study. Visit 1 represented the baseline assessment performed before initiation of metformin therapy. Participants declared no prior metformin use at study inclusion. Samples were obtained using standardized procedures, plasma was separated for analysis and stored under controlled conditions (−80 °C) until analysis.

The baseline characteristics of the study group are presented in Table 1. All participants received dietary counseling at study initiation, and ongoing support from a pharmacist was available throughout the treatment period to address questions related to metformin use and tolerability.

**TABLE 1:** Characteristics of the study group (n = 26). Values are presented as mean ± SD (%CV).

Parameter	S ± SD (%CV)
Age [years]	32.54 ± 6.65 (20.45)
Sex, male/female, n [%]	15 (57.69)/11 (42.31)
Disease duration [years]	7.23 ± 5.22 (72.16)
Body weight [kg]	76.83 ± 13.13 (17.08)
BMI [kg/m^2^]	24.64 ± 3.48 (14.11)
WHR	0.88 ± 0.10 (11.65)
Body fat [%]	23.52 ± 8.71 (37.03)
HbA1c [%; mmol/mol]	7.64; 60.0 ± 1.29 (16.92)
DDI [j/d/kg]	0.71 ± 0.24 (34.04)
TC [mg/dL]	185.64 ± 33.27 (17.92)
Non-HDL [mg/dL]	118.00 ± 26.46 (22.42)
HDL [mg/dL]	65.08 ± 15.19 (23.34)
LDL [mg/dL]	99.91 ± 23.34 (23.36)
TG [mg/dL]	108.87 ± 49.72 (45.67)
AST [U/L]	19.17 ± 6.85 (35.76)
ALT [U/L]	22.61 ± 15.21 (67.25)
AGE	1.88 ± 0.41 (21.92)
VAI	2.72 ± 1.45 (53.38)
TG/HDL	1.75 ± 1.02 (58.21)
eGDR	9.54 ± 2.07 (21.73)
Tobacco use	23%
rtCGM	42.31%
TAR	48.87 ± 24.69 (50.07)
TBR	3.63 ± 3.88 (107.08)
TIR	47.50 ± 21.56 (45.40)
CVD	0%

AGE, Advanced Glycation End-Products; ALT, alanine transaminase; AST, aspartate transaminase; BMI, body mass index; %CV, coefficient of variation; CVD, Cardiovascular Diseases; DDI, daily dose of insulin; eGDR, estimated Glucose Disposal Rate; HbA1c–glycated hemoglobin; HDL, high density lipoprotein; LDL, low-density lipoprotein; non-HDL, non-high-density-lipoprotein; rt-CGM, real-time continuous glucose monitoring; S–arithmetic mean; SD, standard deviation; TC, total cholesterol; TG, triglycerides; TIR, time in range; WHR, waist-hip ratio; VAI, Visceral Adiposity Index.

Participants continued their standard diabetes care throughout the study period, including individualized insulin therapy delivered either by multiple daily injections (MDI) or continuous subcutaneous insulin infusion (CSII).

Participants with type 1 diabetes and features of insulin resistance (Table 1.) were treated with MET (tablets, extended release) at a dose 500–2000 mg/24 h. People were advised to take tablets every evening, after the last meal, and blood samples were collected in the morning. All participants started MET therapy with a dose of 500 mg per day. Due to the variable tolerance of the drug, doses were then individually modified. Some participants continued on a dose of 1,000 mg/day, while people who tolerated MET well could have their dosage increased to 1,500–2000 mg/day. The study design included flexibility in dose adjustments to better reflect actual treatment conditions, including typical gastrointestinal side effects, which often limit the ability to use higher doses. Metformin was administered in the evening, and blood samples collected during follow-up visits were obtained the following morning under routine treatment conditions. Formal adherence assessment tools such as pill counts or validated questionnaires were not used. Plasma metformin concentrations were therefore interpreted as indirect markers of treatment exposure under real-world conditions. Due to the limited group size in this study, analysis of MET concentrations in relation to individual dosage modifications was not conducted at this stage. Differences in drug tolerance remain an important clinical issue that should be addressed in further phases of the study and data analysis.

Several participants received concomitant medications for coexisting conditions, including antihypertensive agents, thyroid hormone replacement therapy, antithyroid treatment, lipid-lowering therapy, antihistamines, and other chronic medications typical for routine clinical care.

### 2.2 Clinical and laboratory assessments

Standard laboratory tests: glycated hemoglobin (HbA1c); lipid profile: high density lipoprotein (HDL), low-density lipoprotein (LDL), non-high-density-lipoprotein (non-HDL), triglycerides (TG); alanine transaminase (ALT), aspartate transaminase (AST); and anthropometric value (body mass, waist and hip measurement, % fat) and advanced glycation end-products (AGE) were performed on all visits. In addition, the body mass index (BMI), waist-hip ratio (WHR), eGDR and visceral adiposity index (VAI) were calculated for each person. VAI was calculated using the following formulas (Amato et al., 2010): For women: VAI = (WC/[36.58 + (1.89 × BMI)]) × (TG/0.81) × (1.52/HDL); For men: VAI = (WC/[39.68 + (1.88 × BMI)]) × (TG/1.03) × (1.31/HDL).

### 2.3 Insulin resistance markers

Insulin resistance was evaluated with indirect markers (eGDR, WHR, BMI, TG/HDL). The eGDR was calculated by the following mathematical formula: 24.31–12.22 (WHR)-3.29 (hypertension 0/1)-0.57 (HbA1c) [mg/kg/min] (Williams et al., 2000). It was assumed that the higher the eGDR, the higher tissue sensitivity for insulin. WHR was checked using the non-elastic measuring tape with the resolution of 0.5 cm and calculated from the following equation: WHR = waist circumference [cm]/hip circumference [cm].

### 2.4 Glucose monitoring

During visits, information from continuous glucose monitoring systems (CGM) or glucometers and daily insulin doses (DDI) were also collected. Glucose monitoring was performed using participants’ routinely used systems from Dexcom (n = 2, 7.7%), Abbott (n = 6, 23.1%), Medtronic (n = 2, 7.7%) CGM systems and other commercially available CGMs (n = 1, 3.9%). More than half of the people did not use CGM (n = 15, 57.7%).

For CGM-derived analyses, at least 14 consecutive days of glucose monitoring data were required. Time in range (TIR) was defined as glucose values between 70 and 180 mg/dL, whereas time below range (TBR) was defined as glucose values below 70 mg/dL, in accordance with international CGM consensus recommendations.

Participants were treated either with multiple daily injections (MDI) using insulin pens (n = 20, 76.9%) or continuous subcutaneous insulin infusion (CSII) systems (n = 6, 23.1%) according to routine clinical practice. Participants used the functional insulin therapy method (FIT).

### 2.5 Determination of metformin in human plasma by UPLC-MS/MS

Metformin concentrations were determined using Ultra-Performance Liquid Chromatography-Tandem Mass Spectrometry (UPLC-MS/MS) method. The method described by the work of Chaudhari et al. (2020) was modified and reported in a separate article (Bekier et al., 2025).

The blank plasma used for method validation was kindly provided by the Regional Blood Donation Centre in Poznań, Poland, and were stored at −80 °C. Plasma samples from patients were prepared by adding 10 µL of each plasma sample, 25 µL of calibration standard solution (or volume compensation), 25 µL of the IS and 40 µL of ACN, each time vortexing for 30 s. The samples were subsequently subjected to centrifugation for a period of 10 min at a speed of 13,000 x g at 4 °C. The obtained supernatant was transferred to inserts and 1 µL was injected into the UPLC-MS/MS system. Concentrations were plotted against the ratio of the peak areas of MET and the internal standard (metformin-D6). Method validation was performed in accordance with the ICH M10 guidelines with respect to linearity, selectivity, accuracy and precision, carryover, recovery, matrix effect and stability, demonstrating appropriate analytical performance and compliance with the acceptance criteria defined by the guideline.

### 2.6 Statistical analysis and artificial neural network modelling

Statistical analyses of metformin concentration (MET), eGDR, AGE, VAI, ALT, AST, TC, LDL, non-HDL, HDL, TG, TG/HDL ratio, body fat percentage, daily insulin dose (DDI), TIR, TBR, HbA1c, BMI, body weight, waist and hip circumference, and WHR were performed for the entire study population as well as for subgroups with lower (<1,181.9 ng/mL) and higher (≥1,181.9 ng/mL) mean plasma metformin concentrations. Subgroups were defined according to the median value of mean metformin concentration. Due to the lack of established plasma metformin concentration thresholds for adults with type 1 diabetes, the median concentration was used as a reference value for assessing potential exposure-response relationships.

Descriptive statistics were presented as median and interquartile range (IQR; Q1–Q3). Analyses were conducted for baseline measurements (visit 1, V1) and for longitudinal comparisons across visits after 6 and 12 months of therapy (V1 vs. V5 vs. V6).

The Shapiro–Wilk test was used to assess normality of data distribution. As the data did not meet the assumptions required for classical parametric analysis, a nonparametric Aligned Rank Transform (ART) analysis of variance was applied (Wobbrock et al., 2011). The ART mixed-effects model included the fixed effects of time (visit), metformin concentration group, and their interaction, with participant identifier included as a random intercept to account for repeated measurements within individuals.

Type III Wald F-tests with Kenward–Roger degrees of freedom correction were used for inference. Pairwise *post hoc* comparisons were performed using estimated marginal means derived from ART models. Comparisons between metformin concentration groups were evaluated within each time point. In the presence of a statistically significant main effect of time, additional pairwise comparisons between visits were performed for the entire cohort and separately within each metformin concentration subgroup. When a statistically significant interaction between time and metformin concentration group was detected, full *post hoc* comparisons across all interaction-defined subgroups were conducted. P-values from multiple comparisons were adjusted using the Benjamini–Hochberg (BH) procedure.

In addition, a sensitivity analysis was conducted after excluding 4 participants who, during visit 1, were found to have a baseline metformin concentration, even though they reported no prior exposure to the drug. The purpose of this analysis was to provide an additional assessment of the relationship between exposure and response in a longitudinal context.

An additional statistical analysis was also performed using an artificial neural network (ANN). The applied ANN consisted of an input layer, one hidden layer, and an output layer. The network was trained using a supervised feed-forward backpropagation algorithm. The input layer contained 23 neurons. The following independent variables were analyzed: WHR, BMI, TIR, TBR, DDI, TC, LDL, HDL, non-HDL, TG, TG/HDL, AST, VAI, AGE, smoking, waist, hip, mean metformin level, duration of the disease, sex, and adipose tissue. The output layer contained one neuron, which was the eGDR. The network was trained until the mean square error took the lowest value. The data were divided into training, testing, and validation sets. The generated ANNs were evaluated based on correlation coefficients and error values calculated separately for each dataset subset. To reduce the risk of overfitting, the network performance was monitored using testing and validation datasets, and the training process was stopped when the minimum error value was achieved. The generated ANNs were evaluated based on their correlation coefficient for training set and value of the error. The correlation coefficient should approach value 1.0000, and the error should take the lowest value.

## 3 Results

### 3.1 Analysis of metformin concentration in blood

**FIGURE 1:**
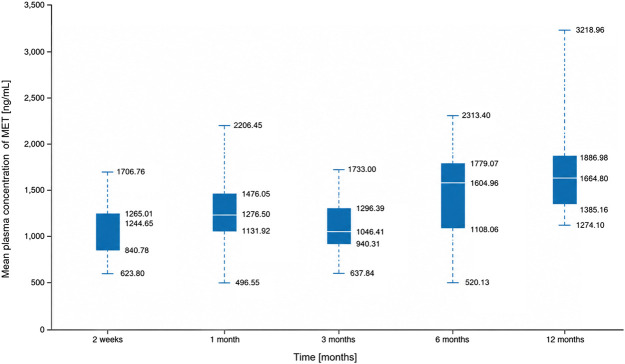
Plasma concentration-time profiles of metformin in adults with type 1 diabetes.

The mean blood metformin concentrations are shown in Figure 1.

#### 3.1.1 Changes between baseline and 12 months (V1 vs. V6)

TIR increased descriptively during follow-up, from 51% (IQR 30–60) at V1 to 55% (IQR 42–66) at V6. No significant overall differences in TIR were observed either between the metformin concentration subgroups or across time points. A significant interaction between metformin concentration subgroup and time was identified (p = 0.0151). In the MET ≥1,181.9 ng/mL subgroup, TIR increased from 47.5% (IQR 30.75–54.5) at V1 to 55.5% (IQR 46.25–68.25) at V6, whereas in the MET <1,181.9 ng/mL subgroup TIR remained relatively stable: 53% (IQR 30–72) at V1 and 55% (IQR 42–61) at V6. Nevertheless, *post hoc* analyses did not confirm statistically significant differences between individual subgroups–time comparisons after correction for multiple testing.

DDI showed a decreasing trend over time, from 0.65 U/kg (IQR 0.52–0.91) at V1 to 0.57 U/kg (IQR 0.42–0.70) at V6, although the overall effect of time did not reach statistical significance (p = 0.0712). In the MET ≥1,181.9 ng/mL subgroup, DDI decreased from 0.66 U/kg (IQR 0.52–0.70) at V1 to 0.56 U/kg (IQR 0.45–0.68) at V6, whereas in the MET <1,181.9 ng/mL subgroup values remained relatively unchanged: 0.63 U/kg (IQR 0.44–1.06) at V1 and 0.64 U/kg (IQR 0.42–1.02) at V6. No statistically significant subgroup differences or subgroup–time interactions were observed. No statistically significant changes in TBR were observed.

The analysis showed significant reduction in LDL values over time: V1: 100 mg/dL (IQR 74.00–113.56), vs. V6: 83.00 mg/dL (IQR 74.38–87.00) (p = 0.0181). Significant reductions were also observed for non-HDL cholesterol: V1: 118.00 mg/dL (IQR 91.75–131) vs. V6: 98.50 mg/dL (IQR 89.25–107.25) (p = 0.0039). Triglyceride levels decreased from V1: 96.00 mg/dL (IQR 67.6–117.7) to V6: 78.00 mg/dL (IQR 58–95.45), although the overall effect of time did not reach statistical significance (p = 0.0586). No significant differences between the MET <1,181.9 ng/mL and MET ≥1,181.9 ng/mL subgroups were observed for LDL, non-HDL cholesterol, or TG.

No significant changes over time were observed for the TG/HDL ratio, ALT, or AST. Although TG/HDL values decreased from V1: 1.44 (IQR 1.16–1.51) vs. V6: 1.09 (IQR 0.76–1.71), the overall effect of time was not statistically significant (p = 0.4495). Similarly, ALT values changed from V1: 15.00 U/L (IQR 14.00–20.00) vs. V6: 16.00 U/L (IQR 12.00–19.00) without statistical significance (p = 0.8842). A significant overall effect of time was observed for VAI (p = 0.0068). Post-hoc analysis demonstrated a significant difference between V1 and V5 (p = 0.0054), whereas comparisons between V1 and V6 (p = 0.0830) and between V5 and V6 (p = 0.1542) were not statistically significant. No significant differences between the MET <1,181.9 ng/mL and MET ≥1,181.9 ng/mL subgroups were observed for TG/HDL ratio, liver enzymes, or VAI.

#### 3.1.2 Changes between baseline, 6 and 12 months (V1 vs. V5 vs. V6)

A statistically significant increase in plasma metformin concentrations was observed during the study (p = 0.0139). No statistically significant differences between the MET <1,181.9 ng/mL and MET ≥1,181.9 ng/mL subgroups were observed.

Statistically significant differences over time were demonstrated for DDI expressed as U/day (p = 0.0176). Median DDI values decreased from 50 U/day (IQR 41–63) at V1 to 40 U/day (IQR 35–50) at V5 and remained at 40.1 U/day (IQR 38–60) at V6. Post-hoc analysis demonstrated a significant difference between V1 and V5 (p = 0.0150), whereas comparisons between V1 and V6 and between V5 and V6 were not statistically significant. No statistically significant differences were found in the MET <1,181.9 ng/mL and ≥1,181.9 ng/mL subgroups.

A decrease in LDL values was also observed, V1: 100.00 mg/dL (IQR 74.00–113.56) vs. V5: 76.00 mg/dL (IQR 64.12–111.00) vs. V6: 83.00 mg/dL (IQR 74.38–87.00) (p = 0.0136). Post-hoc analysis showed significant differences between V1 and V5 (p = 0.0181) and between V1 and V6 (p = 0.0181), while no difference was observed between V5 and V6 (p = 0.9087).

Significant changes over time were also observed for non-HDL cholesterol: V1: 118.00 mg/dL (IQR 91.75–131.00) vs. V5: 96.50 mg/dL (IQR 78.5–117.25) vs. V6: 98.50 mg/dL (IQR 89.25–107.25) (p = 0.0039). Post-hoc analysis demonstrated significant differences between V1 and V5 (p = 0.0059) and between V1 and V6 (p = 0.0059), with no significant difference between V5 and V6 (p = 0.9180).

TG levels decreased, V1: 96.00 mg/dL (IQR 67.6–117.1) vs. V5: 80.00 mg/dL (IQR 61.00–102.5) vs. V6: 78.00 mg/dL (IQR 58.00–95.45); however, the overall effect of time did not reach statistical significance (p = 0.0586). No statistically significant differences between the MET <1,181.9 ng/mL and MET ≥1,181.9 ng/mL subgroups were observed for LDL, non-HDL cholesterol, or TG.

No statistically significant changes over time were observed for ALT, AST, TG/HDL ratio, BMI, WHR, TC, HDL, HbA1c.

Significant changes over time were also observed for AGE levels. The values were as follows: V1: 1.7 (IQR 1.5–2.2) to V5: 1.8 (IQR 1.7–2.2) and V6: 1.9 (IQR 1.6–2.2) (p = 0.0155). Post-hoc analysis demonstrated significant differences between V1 and V5 (p = 0.0248) and between V1 and V6 (p = 0.0248), whereas no significant difference was observed between V5 and V6 (p = 0.7666). No statistically significant differences between the MET <1,181.9 ng/mL and MET ≥1,181.9 ng/mL subgroups were found.

A significant overall effect of time was observed for eGDR. The values were as follows: V1: 9.54 (IQR 8.42–10.04) to V5: 10.90 (IQR 9.89–15.84) and V6: 9.61 (IQR 8.52–10.58) (p = 0.0001). Post-hoc analysis demonstrated significant differences between V1 and V5 (p = 0.0002) and between V5 and V6 (p = 0.0013), whereas no significant difference was observed between V1 and V6 (p = 0.4219). No statistically significant differences between the MET <1,181.9 ng/mL and MET ≥1,181.9 ng/mL subgroups were found.

### 3.2 Artificial neural network (ANN)

**FIGURE 2:**
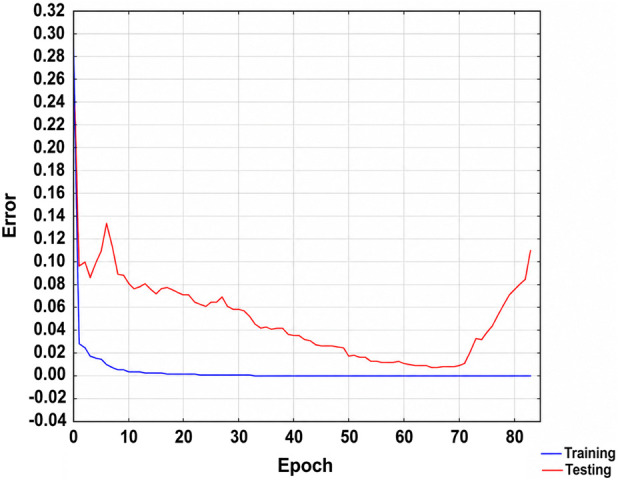
The chart of the learning curve for eGDR.

Due to the limited sample size (n = 26), the ANN analysis was performed only as an exploratory approach, complementary to other methods. The best performance for eGDR was observed for 13 neurons in the hidden layer MLP 23-13-1 with the activation logistic and exponential functions in the hidden and the output layer, respectively. The correlation coefficients were 0.9997, 0.9864, and 0.9812 for the training, testing, and validation sets, respectively. The optimal network was obtained at the 66th training epoch. The learning curve is presented in Figure 2.

The results of the sensitivity analysis are presented in Table 2.

**TABLE 2:** Results of the sensitivity analysis.

Factor	Value
Smoking	8.66
Total cholesterol	4.46
Hip circumference	3.51
Waist circumference	2.87
Non - HDL	2.46
BMI	2.44
Mean metformin level	2.36
HDL	2.25
WHR	2.21
LDL	1.65
TBR	1.54
TIR	1.47
TG/HDL	1.27
Duration of disease	1.23
AGE	1.18
DDI	1.17
VAI	1.14
Adipose tissue	1.12
Sex	1.0000
TG	0.99
AST	0.89

The sensitivity analysis conducted for the ANN model predicting the eGDR revealed varying degrees of influence among the examined variables. Sensitivity values represent the relative impact of each predictor on the model output. Analysis was conducted to assess the relative contribution of predictors within the model; results should be interpreted cautiously due to the risk of overfitting in small datasets. Smoking was identified as the most significant factor, with the highest sensitivity value (8.66), underscoring its critical role in glucose metabolism and insulin sensitivity (Eliasson, 2003). Total cholesterol (4.46) and hip circumference (3.51) were also highly influential predictors. Anthropometric parameters, lipid profile components, mean metformin concentration, and glycemic control indicators (TBR, TIR) demonstrated moderate influence. Age, disease duration, drug–drug interactions, VAI, adipose tissue, and sex exhibited lower effects on eGDR estimation. These findings are exploratory and hypothesis-generating and should be regarded as supportive material complementing the main statistical analyses rather than as confirmatory evidence.

## 4 Discussion

This study provides a comprehensive, real-world assessment of metformin adjunctive therapy in adults with T1DM, combining long-term assessment of plasma metformin concentrations with advanced markers of metabolic control and insulin sensitivity, including TIR and eGDR. To our knowledge, this is the first study to assess the effectiveness of metformin treatment in T1DM based on actual blood metformin concentrations during 1 year of drug therapy.

A meta-analysis by Liu et al. (2015) showed a reduction in body weight, total cholesterol (TC), LDL, HDL levels, and total DDI after metformin treatment in adults with T1D, but only one (Lund et al., 2008) of the 8 studies included in the meta-analysis were conducted for 1 year (Lund et al., 2008; Polskie Towarzystwo Diabetologiczne, 2025). In the other studies, people received treatment for a period shorter than 1 year. Conclusions from long-term observation are provided by the REMOVAL study (Petrie et al., 2017), whose authors suggest that metformin may have a wider role in cardiovascular risk management in adults with T1D. However, the results of this study indicate that in adults with type 1 diabetes, metformin does not improve long-term glycemic control or lead to a permanent reduction in insulin requirements.

The positive benefits of metformin use were not associated with improved glycemic control or an increased risk of hypoglycemia or diabetic ketoacidosis (DKA) (Petrie et al., 2017; Liu et al., 2015). On the one hand, metformin appears to be a reasonable choice for people with double diabetes due to its ability to reduce insulin resistance, its beneficial effects on the cardiovascular system, and its safety. On the other hand, long-term studies do not confirm all the metabolic benefits that may accompany the use of the drug. Although the REMOVAL study is based on a 3-year observation, participants took the drug or placebo at home without monitoring blood drug levels, and visits took place after 3 months and then once a year. More frequent follow-up and monitoring of metformin plasma levels may potentially improve adherence and enhance clinical effects.

In this context, it should be emphasized that traditional markers of metabolic control, such as HbA1c or fasting plasma glucose, may not fully reflect changes in insulin sensitivity, particularly in adults with type 1 diabetes and coexisting insulin resistance. HbA1c represents an average glycemic exposure and does not capture glycemic variability, postprandial excursions, or improvements in time spent within target glucose ranges. Data obtained through CGM, especially TIR, have become useful indicators of short- and medium-term metabolic control and are accepted as clinically meaningful outcomes (American Diabetes Association, 2024; Polskie Towar zystwo Diabetologiczne, 2025; European Association for the Study of Diabetes EASD, 2025).

### 4.1 Metformin exposure and metabolic response

The main finding from this study is that there is substantial interindividual variability in metformin tolerance, and so there is variation in plasma metformin levels. This confirms what we have seen in clinical practice, which is that tolerance, adherence, and individual pharmacokinetics have a big impact on drug exposure. This variability may partly explain the heterogeneous metabolic responses described in previous studies and meta-analyses, which were primarily based on prescribed metformin dose rather than measured systemic exposure. Unlike studies in which metformin was administered for a short period of time without monitoring drug concentrations and with relatively infrequent follow-up visits, our study suggests a possible relationship between systemic metformin exposure and metabolic response. The longitudinal assessment of plasma metformin concentrations allowed characterization of systemic exposure during adjunctive therapy. However, no statistically significant differences between metformin concentration subgroups were observed for the analyzed metabolic parameters. Although plasma metformin concentrations increased during follow-up, the observed metabolic improvements were not associated with statistically significant differences between metformin concentration subgroups. These findings suggest that the relationship between systemic metformin exposure and metabolic response in adults with T1DM may be more complex than previously assumed and requires further investigation in larger controlled studies.

### 4.2 Insulin sensitivity and mean blood glucose levels

Consistent with previous long-term studies, we did not observe a sustained improvement in HbA1c over the 1-year follow-up period. However, our results support the increasingly popular concept that HbA1c alone may be insufficient to capture the metabolic effects of adjunctive therapies targeting insulin resistance. Descriptive trends toward improved TIR, reduced daily insulin dose, and favorable changes in eGDR were observed despite the absence of significant improvement in HbA1c, highlighting the importance of complementary endpoints in T1DM studies.

The transient increase in eGDR observed at 6 months, followed by a partial attenuation after 1 year, is consistent with the results of the other studies (Petrie et al., 2017), in which early metabolic benefits diminished over time. The attenuation of the effect over time may reflect progressive variability in adherence, tolerability or lifestyle factors. Similar temporal patterns have been observed in previous long-term metformin studies in T1DM, suggesting that maintenance of metabolic benefits may depend on sustained systemic exposure and continued behavioral support.

Significant longitudinal changes were also observed for AGE levels. Although the absolute differences between visits were relatively small, the findings may suggest a potential influence of adjunctive metformin therapy on pathways associated with glycation-related metabolic processes. However, the clinical significance of these changes requires further investigation.

### 4.3 Lipid metabolism and cardiovascular risk markers

Metformin therapy was associated with significant improvements in lipid parameters, including LDL and non-HDL cholesterol over time. Significant reductions were observed for LDL and non-HDL cholesterol, while TG concentrations showed a decreasing trend that did not reach statistical significance. Although a significant overall effect of time was observed for VAI, *post hoc* differences between baseline and 12 months were not statistically significant. These results may suggest a moderate effect on cardiometabolic risk that goes beyond glycemic control alone.

No significant longitudinal changes were observed for liver enzyme activity, suggesting that the metabolic effects of metformin observed in the present study were independent of major changes in routine hepatic biomarkers.

### 4.4 The role of eGDR as a clinically relevant endpoint

The study results confirm the value of eGDR as a sensitive marker for assessing the effectiveness of supportive therapies in T1DM. eGDR provides an estimate of insulin sensitivity, which is closely related to cardiovascular and microvascular outcomes. It can significantly complement the efficacy assessment of metformin treatment, especially given its pleiotropic effects beyond metabolic control. The use of HbA1c alone for this purpose may be insufficient.

Exploratory artificial neural network analysis additionally supported the multifactorial nature of insulin resistance in T1DM. Smoking, lipid parameters, anthropometric measurements, and mean metformin concentration were found to be key determinants of eGDR, emphasizing that insulin sensitivity is influenced by a complex interaction of behavioral, metabolic, and pharmacological factors. Importantly, mean metformin concentration was identified as one of the variables contributing to eGDR prediction in the exploratory ANN analysis, suggesting a possible association between systemic metformin exposure and variability in metabolic response.

These findings have important clinical implications. First, they may suggest that the ambiguous effects of metformin in type 1 diabetes described in previous studies may be partly due to insufficient or heterogeneous exposure to the drug, rather than the mechanism of action itself. Second, they emphasize that the benefits of adjunctive metformin therapy may be underestimated if assessed solely on the basis of HbA1c. Finally, they argue for a more individualized approach to adjunctive therapy in type 1 diabetes, including monitoring of insulin sensitivity, indicators obtained from CGM, and potentially plasma metformin concentrations to optimize treatment response.

## 5 Limitations

This study has several limitations, including the relatively small single-center cohort, open-label observational design, absence of a placebo/control group, and limited generalizability of the findings to broader populations of adults with type 1 diabetes. The absence of a control group limits the ability to attribute the observed metabolic changes solely to metformin therapy. Dose adjustment was individualized based on tolerance, which prevented a detailed dose-concentration-response analysis. The study did not include a structured adverse event reporting protocol or formal severity grading of gastrointestinal symptoms. Dose reductions and treatment modifications related to tolerability were managed clinically but were not systematically quantified. Furthermore, the observational nature of the study does not allow conclusions to be drawn regarding causality.

Although the observational, non-randomized design limits causal inference, it reflects real-world clinical practice and allows assessment of metabolic effects of adjunctive metformin therapy under routine treatment conditions, including individualized dosing and variable drug exposure. Importantly, the observed associations were consistent across multiple metabolic endpoints, including eGDR, TIR, lipid parameters and insulin dose, supporting the reliability of the findings despite the study design.

The clinical use of metformin in T1DM is complicated by considerable interindividual variability in treatment response and tolerability. Gastrointestinal adverse effects frequently limit dose escalation, resulting in heterogeneous drug exposure and potentially attenuated metabolic effects. Moreover, improvements in insulin sensitivity may not be adequately reflected by conventional glycemic markers such as HbA1c or fasting glucose, which primarily capture average glycemia rather than underlying metabolic changes (Cleland et al., 2013). As a result, clinicians may perceive the effects of adjunctive metformin therapy as minimal or difficult to interpret, underscoring the need for more sensitive and clinically meaningful indicators of therapeutic response.

The absence of formal adherence assessment limits interpretation of exposure-response relationships. Plasma metformin concentrations should therefore be interpreted as indirect exposure markers rather than definitive adherence measures. Potential confounding effects of lifestyle-related factors, including dietary counseling, physical activity, smoking habits, and other behavioral modifications during follow-up, cannot be excluded and may have independently influenced metabolic outcomes. The age range of female participants was 18–46 years. None of the participants reported the use of hormonal contraception or menopausal hormone therapy among concomitant medications. Information regarding menstrual cycle phase and menopausal status was not systematically collected. Therefore, potential effects of physiological hormonal fluctuations on metabolic outcomes were not evaluated in the present study. Although dietary and lifestyle questionnaires were collected during follow-up, these variables were not included in formal statistical analyses because of the limited sample size and the exploratory, predominantly qualitative nature of the collected data. Missing data and reduced subgroup variability limited inferential statistical analysis for selected variables, including TIR and DDI.

In the present study, ANN modelling was applied as a supplementary exploratory tool and should be interpreted with caution given the limited sample size and the relatively high number of input variables. The sensitivity profile suggested a relatively stronger contribution of smoking, total cholesterol, and selected anthropometric parameters to eGDR estimation. Considering the increased risk of overfitting in small datasets, the ANN findings should be regarded as exploratory and hypothesis-generating rather than predictive, confirmatory, or independent evidence. Validation in larger cohorts is required before any clinical implications can be considered.

## 6 Conclusion

Adjunctive metformin therapy in adults with type 1 diabetes was associated with favorable changes in selected markers of insulin sensitivity and cardiometabolic risk during the observation period. Significant improvements were observed particularly for eGDR, LDL cholesterol, and non-HDL cholesterol, whereas no significant changes were found for HbA1c. Descriptive trends toward improved TIR and reduced daily insulin dose were also observed. Post-hoc analyses did not confirm statistically significant differences between metformin concentration subgroups or individual time-point comparisons for TIR. Plasma metformin concentrations increased significantly during the observation period. No statistically significant differences in treatment response were observed between the subgroups with low and high metformin concentrations. Further controlled studies are needed to clarify the clinical relevance of systemic metformin exposure during adjunctive therapy in T1DM.

This study is reported in accordance with the STROBE guidelines for observational studies.

## Data Availability

The raw data supporting the conclusions of this article will be made available by the authors, without undue reservation.
