# Supplementary material for: Plasma metformin concentration as a determinant of metabolic response in adults with type 1 diabetes: a 1-year prospective observational study
Source: Front Pharmacol. 2026 Jun 30;17:1854936. doi: 10.3389/fphar.2026.1854936 (PMC13364628; doi:10.3389/fphar.2026.1854936)
Supplement: Supplementary file 1 [file Supplementaryfile1.docx]

**METFORMIN DIETARY QUESTIONNAIRES**

QUESTIONNAIRE I – BASELINE

Preliminary information / Lifestyle

1. Do you engage in regular physical activity?
Yes / No

1A. If yes, how often?
1B. What type?

2. Do you smoke cigarettes?
Yes / No

3. Have you ever smoked cigarettes?
Yes / No

4. How many cigarettes per day?

5. Do you take dietary supplements?
Yes / No

5A. If yes, which ones?

Dietary interview

1. Have you changed your eating habits after starting Metformin treatment?
Yes / No

2. Number of meals per day – BEFORE starting Metformin treatment
1 meal / 2 meals / 3 meals / 4 meals / 5 meals or more

3. Frequency of snacking between meals – BEFORE Metformin treatment
Never / 1–3 times per month / Once a week / Several times a week / Once a day / Several times a day

4. Type of bread most often consumed (multiple answers possible) – BEFORE Metformin treatment
White wheat bread
Whole-grain bread (wholemeal/rye/oat/buckwheat)
Refined flour rolls
Pumpernickel
Other (baguettes, croissants, sweet pastries, milk bread)

5. Frequency of whole-grain bread consumption – BEFORE Metformin treatment
Never / 1–3 times per month / Once a week / Several times a week / Once a day / Several times a day

6. Types of cereal products most often consumed – BEFORE Metformin treatment
Whole-grain products (wholemeal flour, coarse groats, brown rice)
Refined grain products (white flour, fine groats, white rice)
Sweetened breakfast cereals / cornflakes

7. Frequency of milk and dairy consumption – BEFORE Metformin treatment
Never / 1–3 times per month / Once a week / Several times a week / Once a day / Several times a day

8. Most frequently consumed dairy products – BEFORE Metformin treatment
Natural yogurt/kefir/buttermilk
Fruit yogurt/kefir/buttermilk
Cow’s milk 0–1.5% fat
Cow’s milk 2–3.2% fat
Plant-based milks
Flavored milks
Cottage cheese
Yellow cheese

9. Frequency of meat consumption – BEFORE Metformin treatment
Never / 1–3 times per month / Once a week / Several times a week / Once a day / Several times a day

10. Type of meat most often consumed – BEFORE Metformin treatment
White meat
Red meat
Both

11. Frequency of fish consumption – BEFORE Metformin treatment
Never / 1–3 times per month / Once a week / Several times a week / Once a day / Several times a day

12. Cooking methods most often used – BEFORE Metformin treatment
Frying / Stewing / Steaming / Boiling / Baking without fat / Grilling

13. Fat used for frying – BEFORE Metformin treatment
None / Various depending on dish / Vegetable oil (incl. olive oil) / Margarine / Butter / Lard

14. Fat used for spreading bread – BEFORE Metformin treatment
None / Various / Mayonnaise / Margarine / Butter / Fat spread

15. Frequency of legume consumption – BEFORE Metformin treatment
Never / 1–3 times per month / Once a week / Several times a week / Once a day / Several times a day

16. Frequency of fruit consumption – BEFORE Metformin treatment
Never / 1–3 times per month / Once a week / Several times a week / Once a day / Several times a day

17. Types of fruit most often consumed – BEFORE Metformin treatment
Apples / Bananas / Berries / Dried fruits / Fruit in syrup / Fruit fried in sugar

18. Number of vegetable servings per day – BEFORE Metformin treatment
None / 1 / 2 / 3 / 4 / 5 or more

19. Frequency of sweets consumption – BEFORE Metformin treatment
Never / 1–3 times per month / Once a week / Several times a week / Once a day / Several times a day

20. Do you use sugar substitutes – BEFORE Metformin treatment?
Yes / No

21. Which sugar substitutes do you use?
Xylitol / Erythritol / Stevia / Cyclamate / Saccharin

22. Frequency of fruit juice consumption – BEFORE Metformin treatment
Never / 1–3 times per month / Once a week / Several times a week / Once a day / Several times a day

23. Frequency of fast food consumption – BEFORE Metformin treatment
Never / 1–3 times per month / Once a week / Several times a week / Once a day / Several times a day

24. Frequency of salty snack consumption – BEFORE Metformin treatment
Never / 1–3 times per month / Once a week / Several times a week / Once a day / Several times a day

25. Frequency of sweetened carbonated drink consumption – BEFORE Metformin treatment
Never / 1–3 times per month / Once a week / Several times a week / Once a day / Several times a day

26. Frequency of energy drink consumption – BEFORE Metformin treatment
Never / 1–3 times per month / Once a week / Several times a week / Once a day / Several times a day

27. Frequency of alcohol consumption – BEFORE Metformin treatment
Never / 1–3 times per month / Once a week / Several times a week / Once a day / Several times a day

28. Type of alcohol most often consumed
Beer / Vodka / Sweet or semi-sweet wine / Dry wine / Liqueurs / Whisky


QUESTIONNAIRE II – AFTER 3 MONTHS

(Questions identical to baseline, referring to current habits)


QUESTIONNAIRE III – AFTER 12 MONTHS

(Questions identical to baseline, referring to current habits)
