# Supplementary figures and images for: Plasma metformin concentration as a determinant of metabolic response in adults with type 1 diabetes: a 1-year prospective observational study
Source: Front Pharmacol. 2026 Jun 30;17:1854936. doi: 10.3389/fphar.2026.1854936 (PMC13364628; doi:10.3389/fphar.2026.1854936)

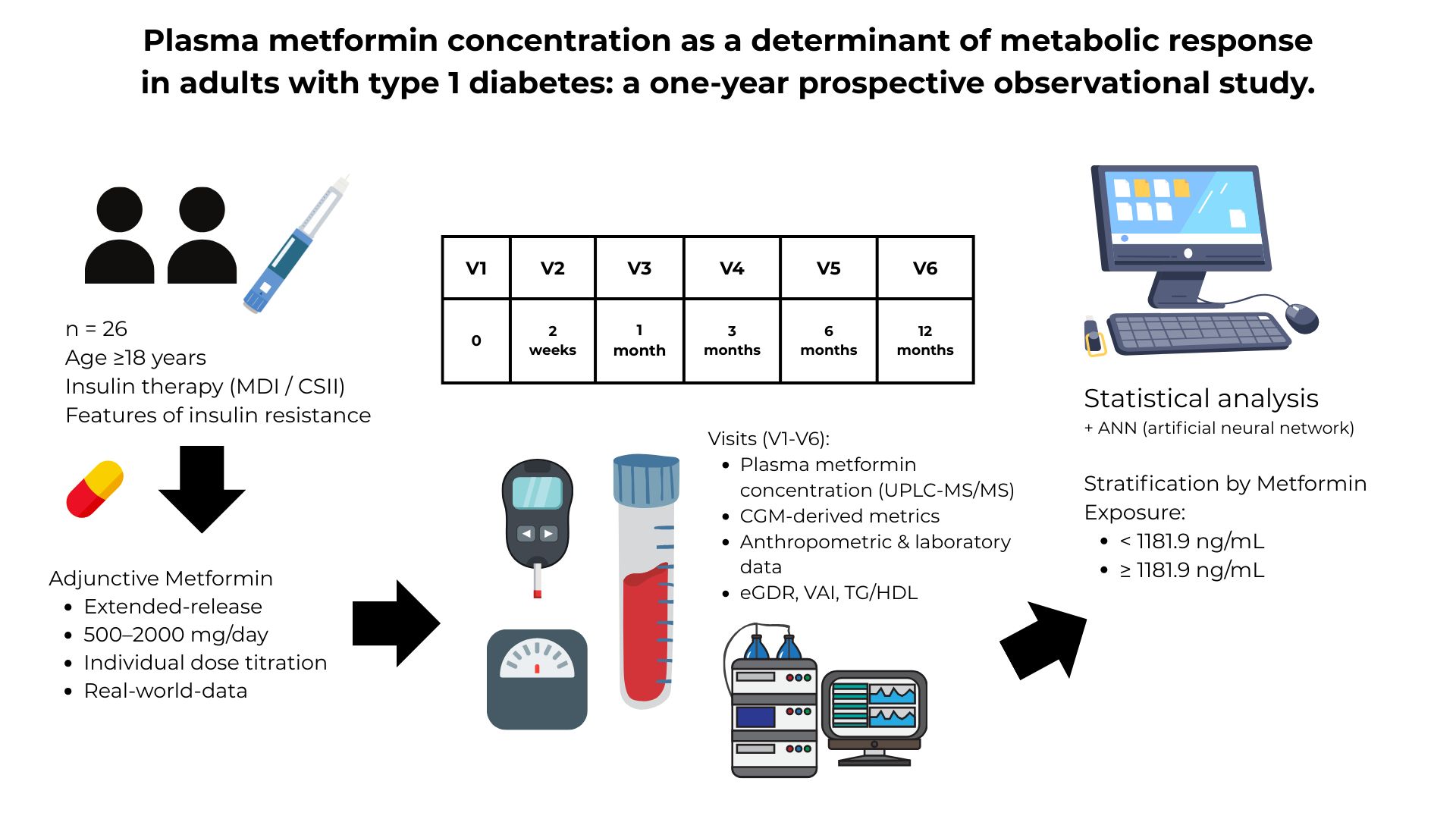

Supplement: Supplementary file 2 [file Image1.JPEG]
